# Pharmacological Treatment for Pregnant Women who Smoke Cigarettes

**DOI:** 10.1186/1617-9625-1-3-165

**Published:** 2003-09-15

**Authors:** BC Chan, G Koren

**Affiliations:** 1 The Motherisk Program, Division of Clinical Pharmacology and Toxicology, The Hospital for Sick Children, University of Toronto, Toronto, Ontario, Canada

## Abstract

Smoking has been associated with several concerns in pregnancy including miscarriage, preterm delivery and stillbirth. Unfortunately, approximately 12% of the pregnant population continue to smoke cigarettes, suggesting a need for additional therapy beyond behavioural change. This paper reviews the literature on the use of nicotine replacement therapy and bupropion (Zyban^®^) in the pregnant human population, the pharmacokinetics of nicotine in the pregnant woman, and current guidelines for smoking cessation for pregnant patients. There are currently four studies that have investigated the use of nicotine patch, three for nicotine gum, and registry and preliminary reports for bupropion. These studies did not show any adverse pregnancy outcomes with the use of pharmacological aid for smoking cessation. All the nicotine replacement therapy studies, with the exception of one randomized-controlled nicotine patch trial had small sample sizes and looked at short-term use of drug in the third trimester. Two studies have examined the pharmacokinetics of nicotine in the pregnant woman. The results from these studies reveal greater nicotine metabolism in pregnant individuals who continue to smoke during pregnancy. Current guidelines from several organizations uniformly recommend that Nicotine Replacement Therapy should be considered if non-pharmacological therapies have been unsuccessful. Bupropion is recommended in pregnancy if the benefits outweigh the risks. There is a need for further studies on the safety and effectiveness of Nicotine Replacement therapy and bupropion in pregnancy. However, considering the current research and guidelines, pharmacological cessation aids should be considered if non-pharmacological therapies have not been effective.

## Introduction

Smoking during pregnancy is a serious teratogenic concern. As early as the 1960s, research revealed a relationship between smoking and birth-weight [1]. Since then, numerous studies have found an association between smoking and an increased risk of several other adverse effects in pregnancy (Table 1). From this information, the general population has been well informed that smoking during pregnancy is a risky behaviour. In a 1999 Annenberg Tobacco Survey given to 14- to 22-year-olds in the US, 97% of non-smokers and 93% of smokers identified that smoking while pregnant can harm the baby [2]. This risk was more widely identified in the survey than deaths due to second-hand smoke and smoking causing lung damage after a few years. Despite the risk of smoking in pregnancy being well established in society, many pregnant women continue to smoke. The National Vital Statistics Report in the US reported that 12.2 percent of all women giving birth in 2000 reported smoking during pregnancy [3]. With 4 million births in the US for the same year, approximately 500,000 pregnancies were at risk because of maternal smoking. In the non-pregnant population, smoking cessation using nicotine replacement therapy (NRT), in the form of gum, patch, lozenge, nasal spray and inhaler have been found to be efficacious in increasing quit rates [4-19]. As a result, NRT has been an acceptable aid for smoking cessation in the general population.

**Table 1:** Reproductive concerns that are associated with cigarette smoke exposure

Ectopic Pregnancy 40–47
Premature Rupture of Membrane 46;48–53
Abruptio Placentae 46;54–63
Spontaneous Abortion 64–68
Placenta Previa 55;69–78
Low birthweight (<2500 g)/Small for Gestational
Age 79–105
Preterm Delivery (<37 weeks) 106–111
Stillbirth 112–115
Neonatal mortality 116;117
Perinatal mortality 105
SIDS 118;119
Cognitive deficit 120–122

The drug manufacturers, on the other hand, have contraindicated the use of NRT in the pregnant population. In a survey of six Boston, Massachusetts, obstetric clinics from 1996–1997, 42% of the obstetric providers did not discuss nicotine replacement therapy with any of their patients who continued to smoke cigarettes [20]. Given a hypothetical scenario about a 20-cigarettes-per-day smoking pregnant woman motivated to quit using nicotine replacement therapy, 44% of the obstetric providers considered it usual practice to prescribe or recommend NRT [20]. However, if the safety of nicotine replacement therapy use in pregnancy was supported by "definitive evidence," the number of practitioners recommending or prescribing this therapy increased to 92%. This stresses the importance of understanding the safety and effectiveness of NRT use in pregnancy. Currently several studies have looked at the use of nicotine patch and nicotine gum in pregnant individuals.

## Nicotine Replacement Therapies and Pregnancy

### Nicotine Patch

Currently four studies have been conducted on the use of nicotine patch in pregnant women. A prospective study by Wright et al (n = 6) [21], monitored the mother and fetus during and after a six-hour application of 21-mg transdermal nicotine patch, following a day of smoking abstinence. Another prospective controlled study by Ogburn et al (n = 21) [22], assessed maternal and fetal effects during a four-day application of 22-mg transdermal patch. Baseline measurements in normal smoking conditions were conducted within a week of patch application. Both studies did not find significant changes in fetal heart rate and umbilical artery Doppler examination readings. The participants in both studies smoked between 10 to 50 cigarettes per day during their pregnancy. A more recent study also enrolled 21 pregnant women smoking more than 15 cigarettes per day [23]. Participants were given 22-mg nicotine patches (24 hours/day) and monitored for four days in-hospital followed by an eight-week period outside the hospital setting. In all patients, non-stress tests were reactive or became reassuring with observation. There were also no significant pre-term deliveries in this study (gestational age ranged between 36.3 to 41.1 weeks). In all three studies, participants were enrolled in the third trimester, so results are limited to the later part of pregnancy. The forth, and only randomized controlled study investigating nicotine patch and pregnancy (n = 250) was published by Wisborg et al [24]. Healthy pregnant women less than 22 weeks pregnant, smoking more than 10 cigarettes per day were given prenatal smoking cessation counseling by a midwife four times during the pregnancy and assigned either nicotine patch or placebo. Treatment duration was 11 weeks for both groups. In the active group, participants were given 15-mg patches (16 hours/day) for eight weeks followed by 10-mg patches (16 hours/day) for three weeks. In this Danish study, the mean birth weight of the newborns was 186 g (95% CI) heavier than the control group. The investigators also found no significance in the rate of low birth weight (less than 2500 g) and preterm babies between groups. Of the participants in the nicotine patch group, 26% had stopped smoking throughout the pregnancy and 14% remained smoked free at one-year post-partum. Both these results were not significant to the control group.

Another controlled study has also looked at the efficacy of nicotine patch in pregnant women [25]. Kapur et al recruited pregnant women in their second trimester and smoking more than 15 cigarettes per day. Participants were randomized to receive either nicotine transdermal patch or placebo patch for 12 weeks. The active group received 15-mg patches (18 hours/day) for eight weeks, followed by 10-mg patches (18 hours/day) for two weeks, then 5-mg (18 hours/day) for two weeks. Researchers enrolled 30 patients before ending the study prematurely after one patient experienced severe signs of withdrawal and rapid and forceful movements from the fetus. These symptoms subsided after the patient smoked a cigarette. Fetal ultrasound, an obstetrical examination and a nonstress test were normal. Of the individuals participating in the study, 17 received nicotine patch, 13 received placebo. Success rates between active and controller rates were not statistically significant, with only three participants completing their assigned therapy in the active group and no participants in the control group.

### Nicotine Gum

Several studies have looked at the use of nicotine gum in pregnant smokers. Specifically, these studies measured maternal nicotine levels and short-term fetal response to nicotine gum. One of the earliest studies in nicotine and fetal response [26] measured fetal breathing movements in 12 pregnant women before and after chewing nicotine gum (Nicorette^®^). Seven participants received one piece of gum containing 4 mg nicotine, while the other five were given two pieces to take simultaneously. Nicotine levels were obtained from venous blood taken before nicotine gum administration, 30 minutes and 60 minutes after the initiation of chewing. There was a significant decrease in fetal breathing 25 minutes after administration of 8 mg nicotine gum, but slowly recovered to non-significant levels at 35 minutes after administration. Decreased fetal breathing was not significant for participants taking the 4 mg nicotine gum. In a separate study [27] (n = 12), investigators compared maternal and fetal response when participants used 4 mg nicotine gum and when they used placebo gum. Participants were given a chewing gum combination of: one piece nicotine followed by one piece placebo, two nicotine gums one after the other, or two placebo gums. Each participant was given all three combinations on separate days. Maternal heart rate, diastolic and systolic blood pressures increase after nicotine gum use but not placebo gum use. Also, fetal heart rate, aortic blood flow and umbilical venous blood flow were unchanged with nicotine and placebo gum use. A more recent study compared nicotine gum to cigarette smoking in separate groups of pregnant women. For a five-day period, 19 participants received nicotine gum, while 10 continued to smoke. Individuals in the nicotine gum group were given a fixed dosage regimen of at least six pieces of gum a day but no more than 30 pieces per day or two pieces per hour. This study found no difference in maternal heart rate and blood pressure, fetal heart rate and umbilical artery resistance [28]. Lower maternal blood nicotine levels were observed in gum use versus smoking, suggesting that in pregnant women, nicotine gum delivers less nicotine than cigarette smoking. All three studies only enrolled pregnant women in their third trimester and smoking more than 10 cigarettes per day.

## Non-Nicotine Smoking Cessation Therapies and Pregnancy

### Bupropion

Zyban (bupropion) is gaining popularity as an alternative to administering nicotine for smoking cessation. Originally intended to treat depression, one of the side effects of bupropion was smoking cessation. Currently, bupropion is classified as Pregnancy Category B, indicating a negative association with fetal harm in animal studies, but no well-controlled studies in human pregnancies. Teratology tests by the manufacturer have been conducted up to doses of 450 mg/kg in rats and up to 150 mg/kg in rabbits, without impaired fertility or fetal harm. The manufacturer has also assembled a bupropion pregnancy registry to understand the safety of this drug during pregnancy [29]. The most recent interim report, issued in December 2002, covered results from September 1997 to August 2002. As of August 2002, the registry had 668 patients enrolled, with 334 pregnancy outcomes. From the 334 pregnancy outcomes, there were 289 live births, 32 spontaneous abortions, one fetal death and 11 therapeutic abortions. Seven birth defects were reported from the 289 live births and one was reported from the therapeutic abortions. The eight birth defects were specifically: one child with bilateral clubfeet, another child with abnormal aortic valve thickening with secondary mild aortic insufficiency, one with ventricular septal defect, one with trivial valvular pulmonic stenosis, one with Klinefelter's Syndrome but no physical abnormalities, one with congenital heart defect (coarctation) and ventricular septal defect, one premature infant with a thickened heart muscle and one induced abortion with possible Down Syndrome detected in prenatal test. Glaxo-SmithKlein also receive retrospective reports of pregnant women using bupropion and their outcomes. From these reports there have been 11 cases of birth defects. An Advisory Committee overseeing the bupropion registry did not find a common pattern among the prospective reports of birth defects. Of the nine retrospective cases, there were four cardiac-related defects. However, whether the rate of cardiac defects is significantly higher than the general population remains undetermined because the denominator is unknown, and because of the bias found in retrospective reports. Motherisk has an unpublished prospective, on-going study investigating the safety of bupropion use during pregnancy. Currently, there are 101 participants in the study and 97 pregnancy outcomes thus far. Of the pregnancy outcomes, there were 71 live births, 18 miscarriages and eight therapeutic abortions. There are still three pregnancies pending and one participant was lost to follow-up. Of the live births, there were no reports of major malformations. Miscarriage rate, mean birth weight and gestational age at delivery were all found to be non-significant when compared to a control group that consisted of pregnant women who had contacted Motherisk, but were not exposed to any teratogens.

## The Pharmacokinetics of Nicotine During Pregnancy

There are two studies that have looked at the pharmacokinetics of nicotine in pregnant women. In a study by Selby et al, 19 Caucasian pregnant women had blood nicotine and cotinine levels measured [30]. This study group reported an inability to stop smoking during their pregnancy, despite understanding the risks. Levels of blood nicotine were lower than expected for the amount of cigarettes reportedly smoked. Cotinine levels, on the other hand, were higher than the average levels per cigarette in pregnant women. The results suggest an increase in nicotine metabolism in a subgroup of pregnant women who cannot stop smoking. A separate study by Dempsey et al measured levels of nicotine and cotinine in 10 pregnant women after infusion of deuterium labelled nicotine [31]. Women were given one or two 30-minute infusions during pregnancy and one infusion post-partum. The dose of nicotine given (1.0 or 1.5 micrograms/kg/min) was similar to smoking two cigarettes. A comparison of nicotine and cotinine levels during and post pregnancy revealed significant increases in nicotine and cotinine clearance during pregnancy. The authors concluded that all pregnant women have accelerated nicotine metabolism. However, in this study, all participants were active smokers during their pregnancy. The results from this study may not represent the metabolism levels of environmental nicotine in non-smokers and ex-smokers. Further studies are necessary to accurately determine whether an increase in nicotine metabolism is found in all pregnant women or only in pregnant women who can't stop smoking. These two studies were also unable to determine whether the observed increase in metabolism is a result of a larger number of metabolism enzyme CYP3s, or greater hepatic clearance due to the pregnancy. There are still many questions surrounding the pharmacokinetics of nicotine during pregnancy. A better understanding of this area will help health authorities set up proper guidelines for smoking cessation in pregnancy.

## Current Guidelines for Smoking Cessation in Pregnancy

### Nicotine Replacement Therapy

In the 2002 edition of the CPS all brands of nicotine gum and patch are contraindicated during pregnancy. The monograph for Nicotrol^®^, available only in the US, elaborated on the reasoning behind the contraindication, stating tobacco smoke and nicotine through animal studies have been shown to cause fetal harm. Therefore the use of this nicotine product may also cause damage to the human fetus.

Most guidelines on the use of NRT and pregnancy, however, do not agree with such restrictions. The Ontario Medical Association (OMA) in literature published in 1999 have recommended that "NRT should be made available to pregnant women who are unable to quit using non-pharmacological methods [32]. Physicians should closely monitor nicotine dosage to ensure that nicotine levels do not exceed smoking levels. As with other drugs, NRT dosage should be matched to suit the smoker's needs." It was also recommended that "Health Canada's labelling requirements should be modified to include consideration of NRT use among pregnant women." The American Agency for Health Care Policy and Research (AHCPR) has similar recommendations for its clinical practice guideline [33]. It is recommended that due to the risks of smoking to the fetus, pregnant smokers should be given more rigorous psychosocial interventions (e.g. videos, quit packages) that go beyond typical advice to quit. Such smoking cessation interventions should persist throughout the pregnancy to ensure abstinence. However if psychosocial intervention is not successful, pharmacotherapy is recommended as a consideration if the benefits of its use surpass the risks of the treatment and continued smoking. The Royal College of Physicians has also expressed similar recommendations for NRT and pregnancy use in Britain [34]. Currently, both France and Germany permit the use of all forms of NRT under physician advice and supervision, provided non-pharmacological methods have been unsuccessful [35]. The United Kingdom has made similar recommendations for the nicotine gum, lozenge, patch and tablet. However, the nicotine spray and inhaler remain contraindicated in pregnancy [35]. In the publication titled *Women and the Tobacco Epidemic: Challenges in the 21^st ^Century *by the World Health Organization, Richard Windsor also recommends the use of NRT if non-pharmacological methods are unsuccessful [36]. Furthermore, it is suggested that five questions be considered by the physician when making the recommendation of NRT to a pregnant patient:

1. Has the patient been provided "Best Practice" (e.g. videos, information packages) methods yet did not quit?

2. Has the patient reported smoking more than 10 cigarettes per day?

3. Does the patient smoke her first cigarette within the first 60 minutes of getting up?

4. Has the patient indicated that she want to quit?

5. Is the fetus's gestational age less than 20 weeks?

There has been a suggestion that the recommendation to initially treat pregnant smokers with psychosocial interventions before considering NRT may not be beneficial to all patients. McNeill et al, in a critique of current NRT practices, suggests that smoking cessation therapy in pregnant patients will have the greatest impact if a medical professional assessed the patient's situation, earlier on in pregnancy, based on past quit attempts and smoking history [37]. A pregnant woman with a low probability of quitting smoking by non-pharmacological means should not be subjected to psychosocial interventions because of the risk of a failed quit attempt causing decreased motivation to stop and resulting in continual smoking during pregnancy. Therefore, the authors suggest the contraindication of NRT use in pregnancy be removed and medical professionals conduct an early assessment, suggesting NRTs to the pregnant mother if the probability of cessation without it is minimal.

### Bupropion

The monograph for Zyban and Wellbutrin, mention animal studies showing no association between the use of bupropion and fetal harm. However, since animal studies do not necessarily reflect human outcomes and because there is a lack of controlled studies, according to the manufacturer, bupropion should be given to a pregnant woman only if it is necessary.

## Conclusion

There have been several studies published about the use of different pharmacological treatments in pregnant women. Currently, none of these studies have revealed a serious concern with NRT or bupropion use during pregnancy. However, most of these studies have small patient numbers, focus on immediate effects of short term NRT use and are limited to use in the third trimester. Nicotine is associated with possible harm to the fetus so psychosocial/behavioural therapy is ideal. However, if non-pharmacological treatments are ineffective, pregnant patients with the physician's assistance should weigh the pros and cons of NRT use. On the one hand, NRTs contains only nicotine, eliminating the patient's exposure to carbon monoxide and other carcinogens. As well, the plasma nicotine levels obtained from NRT use is within the therapeutic range in pregnant women [38], and all formulations should give a smaller dose of nicotine than smoking [39]. On the other hand, the effectiveness of NRTs in pregnancy is still unknown and nicotine withdrawl may have serious consequences to the fetus. The risk to benefit ratio for NRT use in pregnancy is still inconclusive. However, NRT with close supervision by the physician can potentially satisfy a pregnant woman's desire to smoke without exposing the patient to nicotine levels higher than that obtained from smoking. Currently, research has not shown an association between bupropion use in pregnancy and fetal malformations. However, more research is required before any definite conclusions can be made. Physicians and patients should weigh the risks and benefits of taking this medication during pregnancy.

There is a need for further studies on the safety and effectiveness of NRT use in pregnancy. At the same time, greater effort should be placed in educating medical professionals about smoking cessation therapy for pregnant women, so health care providers can offer the best possible treatment. With better therapy in this specialized group, hundreds of pregnancies with adverse outcomes can be avoided.

## Competing interests

The authors declare that they have no competing interests.
